# *Salmonella* Derby Clonal Spread from Pork

**DOI:** 10.3201/eid1105.041042

**Published:** 2005-05

**Authors:** Sylvia Valdezate, Ana Vidal, Silvia Herrera-León, Javier Pozo, Pedro Rubio, Miguel A. Usera, Ana Carvajal, M. Aurora Echeita

**Affiliations:** * Instituto de Salud Carlos III, Madrid, Spain;; † Universidad de León, León, Spain

**Keywords:** swine, Salmonella enterica Derby, clonal spread, PFGE typing, human food

## Abstract

The genetic diversity of the Derby serotype of *Salmonella enterica* in Spain was examined by pulsed-field gel electrophoresis (PFGE). Out of 24 identified PFGE profiles, a major clone was detected in 19% of strains from humans, 52% from food, and 62% from swine. This clone (clone 1) was isolated from pork products, suggesting swine as its source.

Salmonellosis, a major public health concern worldwide, is one of the most common causes of human gastroenteritis. It is caused by the ingestion of contaminated food because of a failure to control *Salmonella* infection in animal husbandry. Its carriers are swine, poultry, and cattle, along with eggs, milk, and fresh seafood (1). *Salmonella* in pork carcasses is a result of fecal contamination during slaughtering and processing. In this case, the carrier swine is the main initial source of contamination (2,3), and bacteria are usually located in the pharynx, lymph nodes, stomach, and feces (4).

During 2002, *Salmonella enterica* subsp. *enterica* serotype Derby was the second and sixth most common *Salmonella* serotype in clinical and nonclinical nonhuman sources, representing 24.0% and 3.7% of isolates reported to the Centers for Disease Control and Prevention and the National Veterinary Service, respectively (5). The frequency of *S*. Derby among isolates from animals received at the Spanish National Reference Laboratory for *Salmonella* and *Shigella* (LNRSSE) was higher than in previous years because of isolates received from swine farms. The incidence of *S*. Derby in Spain has remained stable in isolates of human and food origin, accounting for 0.55% and 2.5%, respectively, of *Salmonella* recovered from these sources from 1997 to 2003 (M.A. Echeita, unpub. data).

The persistence and clonal survival of *S*. Derby in swine populations and in closed environments and the role of pork and pork products as sources of outbreaks in humans have been described previously (6–8). *S*. Derby is one of the most common serotypes of *Salmonella* in swine. It accounted for 24.2% of the serotypes at 3 nurseries and 9 finishing farms of 2 commercial swine production companies in North Carolina (9) and for 37.1% at 5 swine-finishing units in Quebec, Canada (10).

To obtain epidemiologic insights into human acquisition of the *S*. Derby serotype compared with swine-related strains, antimicrobial susceptibility profiles were obtained and genetic characterization by pulsed-field gel electrophoresis (PFGE) was conducted in strains isolated from humans, food, and the environment. Unlike more common serotypes such as Enteritidis or Typhimurium, this serotype has not been subjected to intensive molecular epidemiologic studies.

## The Study

A total of 110 *S*. Derby isolates collected during a 3-year period (2000–2002) were studied. Forty-seven of these isolates (LNRSSE group) were submitted by 34 Spanish laboratories and had the following origins: humans (n = 16), food (n = 23), ill animals (n = 6), and environmental sources (n = 2), as shown in Table 1. The remaining 63 isolates (swine group) were obtained from the stools and mesenteric lymph nodes of 48 swine slaughtered at 4 swine operations (A, B, C, and D) and 6 finishing units (units 1–6) (Table 2) in Spain. The swine reared on these farms shared the same feed supply and had similar genetic backgrounds.

**Table 1:** Characteristics of Salmonella enterica Derby serotype isolates (N = 47) from the LNRSSE Group (Spanish National Reference Laboratory for Salmonella and Shigella)*

Specimen	Isolation date	Geographic origin	PFGE profile	Resistance profile	GyrA mutation†
Human origin (16 strains)					
1	9/4/01	Barcelona	19		
2	3/5/02	Lugo	4	T	
3	4/25/02	Madrid	3	S,Su	
4	4/25/02	Unknown	NT	A,S,Su,T,C,Nx	Tyr 83 (>256/0.12)
5	5/23/02	Orense	10	A,S,Su,T,C	
6	7/27/02	Castellón	17		
7	7/27/02	Barcelona	11	A,S,G,To,Ap,Su,T,SxT	
8	7/8/02	Zaragoza	11		
9	8/8/02	Barcelona	1	Su,T,SxT	
10	8/20/02	Tarragona	23		
11	8/22/02	Alicante	16	S,T,Nx	Asn 87 (64/0.09)
12	7/8/02	Oviedo	1	S,Su,T	
13	9/1/02	Zamora	1	S,Su,T	
14	9/30/02	Huesca	16	T	
15	11/28/02	Ciudad Real	23	S,Su,T	
16	11/5/02	Caceres	5	A,T	
Food origin (23 strains)					
Unknown	3/22/02	Barcelona	18	S,Su,T,SxT	
Seafood	5/1/00	Salamanca	14	A,Su,T,SxT	
Sausage	5/2/00	Bilbao	1	Su,T	
Pork	6/11/00	Cádiz	7	T	
Pork	6/16/00	Granada	23	Nx	Phe 83 (>256/0.09)
Pork	7/10/00	Zaragoza	1	S,Su,T	
Sausage	6/16/00	Navarra	1	S,Su,T	
Lamb	1/10/01	Málaga	1	A,T	
Minced meat	4/2/01	Toledo	12	S,T	
Unknown	7/4/01	Valencia	12	S,T	
Sausage	7/2/01	Granada	1	S,Su,T	
Minced meat	10/5/00	Toledo	13	T	
Unknown	7/18/00	Vitoria	NT	A	
Corn	12/31/01	Bilbao	NT		
Hamburger	4/16/02	Córdoba	1	Nx	Phe 83 (>256/0.1)
Pork	4/19/02	Jaén	1	S,Su,T	
Sausage	5/28/02	Soria	1	S,Su,T	
Pork	6/14/02	Oviedo	1	S,Su,T	
Pork	6/19/02	Oviedo	1	S,Su,T	
Pork	9/10/02	Madrid	1	T	
Unknown	9/26/02	Burgos	1	Su,T,SxT	
Sausage	10/29/02	Barcelona	24	T	
Pork	9/1/02	Lugo	20	T	
Environmental origin (2 strains)					
Groundwater	9/3/01	Valencia	12	S,T	
Beach	2/29/02	Bilbao	1	Su,T,SxT	
Ill animal origin (6 strains)					
–	–	Soria	14	A,T	
Swine	5/22/02	Toledo	1	S,SuT	
Swine	3/7/02	León	6	S,T	
Swine	3/7/02	León	6	S,T	
Swine	3/6/02	León	6	S,T	
Swine	1/22/02	León	9	Su,T,SxT	

*PFGE, pulsed-field gel electrophoresis; Nx, nalidixic acid; Cp, ciprofloxacin; T, tetracycline; S, streptomycin; Su, sulfamethoxazole; NT, not typeable; A, ampicillin; C, chloramphenicol; G, gentamicin; To, tobramycin; Ap, apramycin; SxT, cotrimoxazole. †MIC for Nx/Cp, μg/mL.

**Table 2:** Salmonella enterica* Derby strains (N = 63) from the swine group

Swine operations/ finishing units	No. of PFGE profiles/resistance patterns	No. of PFGE profiles (no. of swine)	Resistance phenotype (no. of swine)
A-1 (6 strains)	1/2	1 (5)	S,G,To,Su,T,C (1)
			A,S,G,To,Nt,Su,T,Nx,C (4)†
A-2 (3 strains)	1/1	1 (3)	A,S,G,Su,T,C (3)
B-3 (16 strains)	2/7	1 (4)	(1)‡
			S,Su,T (2)
			S,Su,T,C (1)
		8 (11)	(1)§
			(1)‡
			S,Su,T (8)
			A,S,G,To (1)
C-4 (1 strain)	1/1	1 (1)	S,Su,T (1)
D-5 (32 strains)	3/3	1 (17)	S,Su,T (17)
		2 (2)	S,Su,T (2)
		21 (1)	A,S,Su,T,SxT (1)
D-6 (5 strains)	2/4	22 (2)	A,S,G,T,Ap,Su,SxT,C (1)
			A,S,G,To,Ak,Su,T,SxT,C (1)
		15 (2)	T (1)
			A,S,G,To,T (1)

*Identified in 48 swine slaughtered at 4 swine operations (A–D) and 6 finishing units (1-6) in Spain. PFGE, pulsed-field gel electrophoresis; S, streptomycin; G, gentamicin; To, tobramycin; Su, sulfamethoxazole; T, tetracycline; C, chloramphenicol; A, ampicillin; Nt, netilmicin; SxT, cotrimoxazole. †GyrA mutation (MIC nalidixic acid/ciprofloxacin): Hys 87 = 48/0.09 μg/mL. ‡Susceptible to all antimicrobial agents tested except spectinomycin. §Susceptible to spectinomycin.

The isolates were screened for antimicrobial resistance by the disk diffusion method (Clinical and Laboratory Standards Institute, Wayne, PA, USA). All isolates were susceptible to amoxicillin-clavulanate, cephalothin, cefuroxime, cefotaxime, ceftriaxone, cefoxitin, imipenem, ciprofloxacin, and enrofloxacin. All strains but 1 were resistant to spectinomycin and had variable resistances to the remaining antimicrobial agents tested (Tables 1 and 2).

A total of 70% and 85.7% of the isolates in the LNRSSE and swine groups were resistant to tetracycline, 36.6% and 85.7% to streptomycin, 33.3% and 71.4% to sulfonamides, 23.3% and 50% to ampicillin and ticarcillin, 16.6% and 42.8% to cotrimoxazole, and 13.3% and 7.1% to nalidixic acid, respectively (Tables 1 and 2). Phenotypes in both groups were most commonly resistant to tetracycline and sulfamethoxazole/tetracycline. Multiple drug resistance (resistance to ≥4 drugs), was observed in 23.3% of LNRSSE isolates and 64.3% of swine isolates.

In addition, the MICs for nalidixic acid and ciprofloxacin were determined in 7 nalidixic acid–resistant strains by the E test (AB Biodisk Dalvagen, Solma, Sweden). The results of these tests are shown in Tables 1 and 2. GyrA, GyrB, ParC, and ParE point mutations in these isolates were genetically characterized (11). The results showed amino acid substitutions only in GyrA: Ser83→ Phe or Tyr and Asp87 → Asn or His (GenBank accession no. AY858891) (Tables 1 and 2).

**Figure 1:**
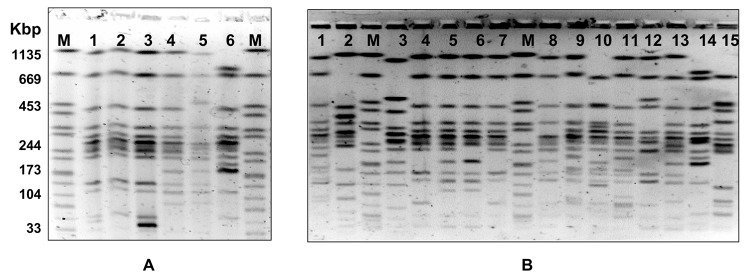
Twenty-one pulsed-field gel electrophoresis profiles identified in Derby strains of *Salmonella enterica.* A) profiles 3, 1, 2, 4, 5, and 8 (lanes 1–6). B) profiles 20, 21, 23, 14, 12, 14, 13, 14, 15, 9, 11, 10, 16, 17, 7, and 19 (lanes 1–15). The control strain (Braenderup serotype) used as a molecular marker in shown in lane M.

**Figure 2:**
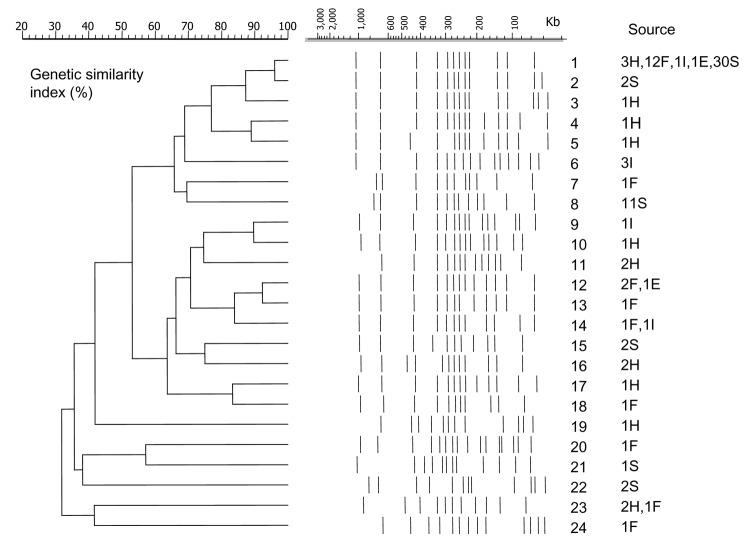
Twenty-four pulsed-field gel electrophoresis profiles and similarity dendrogram of 110 genomic DNAs of Derby strains of *Salmonella enterica* isolated from human stools (H), food (F), ill animals (I), environmental sources (E), and slaughtered swine (S). The number refers to the profile number. The number of strains and their corresponding sources are shown. A DNA molecular weight marker derived from the Braenderup serotype was used as a control.

To define clusters, PFGE was carried out according to the Salm-gene protocol (12) by using the CHEF-DR II System (Bio-Rad Laboratories, Hercules, CA, USA). Fingerprints were compared with GelCompar (Applied Maths, Kortrijk, Belgium). Pattern clustering was performed by using the unweighted pair group method with an arithmetic mean and the Dice coefficient (13) with a tolerance of 0.8%. Each PFGE profile or clone, which differed by at least 1 band from a previously recognized type, was considered a distinct profile. Twenty-four PFGE profiles were observed among the 110 strains (Figure 1). A total of 10, 9, 2, 4, and 6 clones were detected in strains from human, food, environmental, sick animal, and slaughtered swine sources, respectively (Tables 1 and 2). Twelve clones displayed closely related profiles, and paired PFGE profiles differed from each other by 1 band (1-2, 2-3, 4-5, 9-10, 12-13, and 12-14), 2 bands (1-3 and 13-14), or 3 bands (16-18) (Figure 2).

Eleven PFGE profiles (10.0% of the strains) were unique. Conversely, PFGE profile 1 accounted for 52.7% of all isolates studied: 3 from humans, 12 from food, 1 from the environment, 1 from an ill swine, and 42 from 30 slaughtered swine (Tables 1 and 2). PFGE profile 8 represented 10.0% of the characterized strains isolated from 11 swine slaughtered at operation B finishing unit B3. No other PFGE profile accounted for >3% of the strains. In the 63 strains obtained from 48 swine slaughtered, 6 PFGE profiles were observed: 1 (62.5%), 2 (4.2%), 8 (22.9%), 15 (4.2%), 21 (2.8%), and 22 (4.2%).

The corresponding values of genetic similarity ranged from 32% to 96% according to the dendrogram constructed with the different PFGE profiles (Figure 2). Typeability was ≈96.3% because autodigestion occurred in 4 strains. The discriminatory ability calculated by using the Simpson index was 0.9931 for the LNRSSE group, which was selected without a geographic link for any isolates.

## Conclusions

The study was conducted because of the *S*. Derby isolate, detected through *Salmonella* surveillance programs, emerged on several swine farms in Spain. We investigated the genetic diversity of *S*. Derby isolates in these herds by using PFGE to obtain information on their genetic backgrounds.

The predominant clone (clone 1) was identified in 42 strains isolated from 30 animals at 4 swine operations and 5 finishing units. It appeared as a single clone in 3 swine operation finishing units (A-1, A-2, and C-4), or in combination with other PFGE profiles (8 for B-3; 2 and 21 for D-5).

When the persistence of a clone was detected, we tracked its spread from food products to humans by testing *S*. Derby strains isolated from humans, food, the environment, and ill animals in different areas of Spain. Clone 1 was identified in 18.7% of human strains, 52.2% of food strains, as well as in a strain collected on a beach and in another from an ill animal. Moreover, a closely related clone (clone 2) was detected with clone 1 in the swine operation finishing unit D4. This finding might be the result of these clones sharing a common ancestor. The dissemination of clone 1 may be more widespread because LNRSSE group isolates were selected on the basis of no epidemiologic links and were not associated with national or local food outbreaks.

Because resistance of *Salmonella* to antimicrobial agents is a worldwide problem, drug susceptibility was also examined. Resistance to amoxicillin-clavulanate, cephalosporins, imipenem, and fluoroquinolones was not detected. However, resistance to tetracycline was high (≈75%), which is similar to that recorded in a study of 304 *S*. Derby swine isolates (9), but differs from that obtained in human and food isolates (49%) (14). In addition, susceptible isolates from the swine group showed increased resistance to other drugs when compared with those in the LNRSSE group. No relationship was observed between the time of isolation of a specific clone and increased resistance.

A novel GyrA mutation (Hys 87) was identified in 4 swine strains of clone 1 that showed resistance to nalidixic acid and low-level resistance to ciprofloxacin. These strains, which also showed resistance to other antimicrobial agents (ampicillin, streptomycin, gentamicin, tobramycin, netilmicin, tetracycline, and sulfamethoxazole), may be attributed to multiple resistance factors present on transmissible genetic elements (15).

In summary, this study detected a clone of *S*. Derby in pork-derived products and subsequently in humans. Our results emphasize the need for safe food-handling practices to limit the intake of raw or undercooked pork, and procedures to avoid colonization of swine herds with *Salmonella*. They also support establishing guidelines to reduce foodborne pathogens on swine farms and in slaughterhouses. Moreover, because of the emergence of bacterial resistance to antimicrobial agents as a result of their extensive use in animal husbandry, measures such as modification of doses of antimicrobial agents, control of *Salmonella* infections in primary production facilities, and effective epidemiologic surveillance must be implemented to determine potential routes and spread of infection.
